# Growth performance and feed utilization of African catfish *Clarias gariepinus* fed a commercial diet and reared in the biofloc system enhanced with probiotic

**DOI:** 10.12688/f1000research.12438.1

**Published:** 2017-08-22

**Authors:** Iskandar Putra, Rusliadi Rusliadi, Muhammad Fauzi, Usman M. Tang, Zainal A. Muchlisin

**Affiliations:** 1 Faculty of Fisheries and Marine Sciences, Universitas Riau, Pekanbaru , Riau , 28293, Indonesia; 2 Faculty of Marine and Fisheries , Syiah Kuala University, Banda Aceh, 23111, Indonesia

**Keywords:** Biofloc, Probiotic Frequency, Survival Rate

## Abstract

**Background**

The objective of the present study was to evaluate the growth performance and feed utilization of African catfish *Clarias gariepinus* fed a commercial diet and reared in the biofloc system enhanced with probiotic.

**Methods**

The treatment was the frequency of probiotic application into the cultured system, namely, 5-day interval, 10-day interval, and 15-day interval for 60 days of experiment. Biofloc culture was grown in an experiment tank (vol. 2000 L) by mixing the probiotic ( *Bacillus* sp.) 10 mL and molasses 200 mL per liter of water.  The fish was stocked into the biofloc system 7 days after cultured at stocking density of 1000 fish tank ^-1^.  The fish was fed a commercial diet that contains 38% crude protein, twice a day at satiation. The application of probiotic was reperformed after 5 days, 10 days, and 15 days after stocking.

**Results**

The study showed that the growth performance, survival, and feed utilization of African catfish were higher in the treatment at 5-day intervals over 60 days. The ANOVA test showed that the application frequency of probiotic into biofloc system of cultured media had the significant effect on the growth performance, survival rate, and feed utilization of African catfish.

**Conclusion**

The best growth performance and feed utilization were  found at the application of probiotic into biofloc system at 5-day intervals over 60 days.

## Introduction

Feed is one of the important agro-inputs in aquaculture production system that contributes to approximately 40–60% of production cost ^1, 2^ and it has direct effect on the growth rate of the fish ^3– 6^. The aquaculture activity is commonly produced waste, for example, feed remains and feces which changes into ammonia and nitrite once the oxygen level is low. In the closed culture system the concentrations of ammonia (NH _3_) and nitrite (NO _2_) are increasing rapidly and would be toxic to organisms ^7, 8^.

According to Asaduzzaman *et al.* ^9^ and De Schryver *et al.* ^10^ the intensive application of commercial feed in the aquaculture causes environmental pollution and increases the possibility of the disease outbreak. Therefore, the water quality management is crucial in the aquaculture system. The objective of water quality management is to provide the comfortable environment and meet the optimum requirements for cultured organisms ^11^. According to Gunadi and Hafsaridewi ^12^ the microbial activities can be used to improve water quality and reduce the burden of contamination by fish farming waste. Therefore, the heterotrophic bacteria have promising potency to be applied in the utilization of waste ammonia in the fish culture. Beside, these bacteria are formed as a floc (clumps) in the cultures media; hence it can be used as an alternative feed source for cultured fish ^13^. Biofloc has abilities to suppress the toxic compounds such as ammonia and harmful bacteria (pathogenic) so that the cultured organisms grow well ^14^. Application of biofloc in the cultures system has been reported by several researchers, for example, in the culture of channel catfish ^14, 15^, in the South American catfish *Rhamdia quelen* ^16^, in Nile tilapia *Oreochromis niloticus* ^17, 18^, *Farfantepenaeus brasiliensis* ^19^, and in the cultured system of the shrimps *Litopenaeus vannamei* and *Penaeus monodon* ^11, 20^. However, application of biofloc on African catfish *Clarias gariepinus* cultures has never been reported previously.

 African catfish is the popular species for aquaculture business in Southeast Asian countries ^21^. This species has several advantages, for example, resistance to diseases and handling stress and high growth rate ^22^, thus accounting for its commercial importance worldwide ^23^. Nowadays, the fish farmer fed a commercial diet for African catfish. The protein requirement for African catfish ranges from 25% to 40%, lipid 9.5 to 10%, carbohydrates 15 to 30%, vitamins 0.25 to 0.40%, and minerals 1.0% ^1^, with energy level of 2000 cal/g to 3000 cal/g ^24^. In addition, the application of probiotic into African catfish diet has been reported by several researchers, for example, Al-Dohail *et al.* ^25^, Ige ^26^, and Dennis and Uchenna ^27^. However, application of probiotic combing with biofloc has never been reported previously. Hence, the aim of the study was to evaluate the growth performance and feed utilization of African catfish fed experimental diet reared in the biofloc cultured system and enhanced with probiotic.

## Methods

### Site and time

The research was conducted from June 2016 to August 2016 at Aquaculture Technology Laboratory, Faculty of Fishery and Marine Sciences, Riau University, Indonesia. The experiments were carried out within the ethical guidelines provided by the research institution and national or international regulations.

### Experimental design

The completely random design (CRD) method was used in this study. The tested treatment was the frequency of probiotics application (bacteria inoculation), namely, at 5-day interval (treatment A), 10-day interval (treatment B), and 15-day interval (treatment C). The treatment was conducted at three replications. The experimental fish was maintained in the canvas tank (vol. 2000 L) at stocking density of 1000 fishes and reared for 60 days.

### Biofloc culture and feeding

The biofloc was cultured in the nine canvas tanks with a volume of 2000 L. Each tank was filled with water up to a water level of 100 cm or equivalent to 2000 L. Biofloc culture was done by mixing the probiotic ( *Bacillus* sp.) 10 mL and molasses 200 mL L ^-1^ of water and then mixed into the cultures fish tanks and aerated continuously for 7 days to grow the floc.

The catfish larvae were stocked at the density of 1000 fish tank ^-1^ with average weight 1.12±0.05 g and average total length 4.42±0.09 cm. The application of 10 mL inoculants bacteria with density of *Bacillus* sp. about 5×10 ^10^ colony forming units (CFU) was performed according to respective treatment, that is, 5-day, 10-day, and 15-day intervals. The experimental catfish feed was a commercial diet with 38% crude protein, crude lipid 5%, and crude fiber 6%, mineral mix 13%, and 13% moisture contents. The fish were fed twice a day at satiation. The weight gain of fish was measured every 12 -days for 60 days.

### Measured parameters

The weight gain was calculated as follows: W = Wt – Wo, where W is weight gain (g), Wt is the weight of the fish at the end of experiment (g), and Wo is the weight of fish at the start of experiment (g). The daily growth rate, survival rates, and feed utilization were calculated based on Muchlisin *et al.* ^28, 29^ The main water quality parameters such as dissolved oxygen (DO), pH, and temperature were measured using a digital water checker (YSI-550 A, ASTM, Alla, France) at 6-day intervals, while total ammonia nitrogen (TAN) was measured every 6 days using spectrophotometric method ^30^.

### Data analysis

The data were subjective to one-way analysis of variant (ANOVA) test to determine the effect of treatment on the tested parameters and followed by Newman-Keuls multiple range test with a confidence level of 95%, while the water quality of the data was analyzed descriptively.

## Results

The ANOVA test showed that the treatment had a significant effect on the weight gain (WG), specific growth rate and survival rate (SGR), feed efficiency (FE), and feed conversion ratio (FCR) (P<0.05). The study showed that the highest weight gain and specific growth rate were recorded at treatment A; these values were different significantly from other treatments. A similar trend was also found in the survival rate (SR) where the highest survival rate was recorded in treatment A, but this value was not different significantly from treatment C ( Table 1). The highest feed efficiency and lower feed conversion ratio were also found in fish with application of probiotic into biofloc system at 5-day intervals (treatment A). However, these values were not different significantly from treatment C (probiotic application at 15-day intervals). In addition, the water temperature ranges from 29.50°C to 29.62°C, dissolved oxygen rages from 3.64 mg L ^-1^ to 3.88 mg L ^-1^, and pH ranges from 6.93 to 7.02. In addition, the ammonia (NH _3_) content ranged from 0.292 mg L ^-1^ to 0.411 mg L ^-1^ and nitrite content ranged from 0.08 mg L ^-1^ to 0.09 mg L ^-1^. Therefore, there were no significant differences regarding water quality among the treatments; however, the quality in treatment A was slightly better compared to two other treatments ( Table 2). The data showing the total length, body weight and total feed consumed by fish at every experiment can be found in Dataset 1.

**Table 1.** The growth performance, survival rate, and feed utilization of African catfish, *Clarias gariepinus*. Mean of values in the same row followed by a different superscript that are significantly different ( *p*<0.05).

No	Parameter	Application frequency of probiotic		
		5-day interval	10-day interval	15-day interval
1.	Weight gain (g)	125.89±1.96 ^b^	85.57±5.80 ^a^	94.19±22.81 ^a^
2.	SGR (% day ^-1^)	7.91±0.06 ^b^	7.28±0.06 ^a^	7.34±0.40 ^a^
3.	Survival rate (%)	95.77±0.66 ^b^	75.23±9.70 ^a^	91.37±4.78 ^b^
4.	Efficiency of the feed (%)	110.86±2.60 ^b^	88.17±6.89 ^a^	90.98±5.69 ^a^
5.	Feed conversion ratio	0.90±0.02 ^a^	1.14±0.08 ^b^	1.10±0.07 ^b^

**Table 2.** The main water quality parameter of the cultured media of African catfish ( *Clarias gariepinus*).

Parameters	Unit	Application frequency of probiotic		
		5-day interval	10-day interval	15-day interval
Temperature	C°	29.53±1.72 ^a^	29.62±1.82 ^a^	29.5±1.72 ^a^
DO	mg L ^-1^	3.88±0.44 ^a^	3.64±0.32 ^a^	3.64±0.35 ^a^
pH	-	7.02±0.10 ^a^	6.93±0.11 ^a^	6.97±0.09 ^a^
(NH _3_)	mg L ^-1^	0.292±0.11 ^a^	0.332±0.176 ^a^	0.411±0.195 ^b^
NO _2_	mg L ^-1^	0.09±0.076 ^a^	0.08±0.036 ^a^	0.09±0.035 ^a^

Study results The total length, body weight and total feed consumed by fish at every experiment Click here for additional data file. Copyright: © 2017 Putra I et al. 2017 Data associated with the article are available under the terms of the Creative Commons Zero "No rights reserved" data waiver (CC0 1.0 Public domain dedication).

## Discussion

The study showed that the growth performance, survival rate, and feed utilization of African catfish were the highest in the application of probiotic into the biofloc system at 5-day intervals. This was presumably due to the fact that the applications of probiotics every 5-days can maintain the density of bacteria at suitable forms and effectively decompose organic materials well. This is indicated by lower ammonium (NH _3_) content in treatment A. According to Widanarni ^31^ the application of biofloc into culture system can improve water quality and reduce the burden of contamination of fish culture waste in the surrounding waters. In addition Irianto ^32^ stated that *Bacillus* sp. can improve the quality of the cultured media by decomposing organic materials, suppress the growth of pathogenic, and balance the microbial and had a positive effect on fish health and growth. 

Besides maintaining the water quality, biofloc is also playing an important role as alternative natural feed for cultured fish. This is because the biofloc contains crude protein that reached 48–53% ^33, 34^ and therefore the Feed Conversion Ratio (FCR) in treatment A was 0.90 (below 1.00) and the feed efficiency was higher than 100%. This is because of beside fed on the commercial diet the fish was also fed on floc that contain planktons. This value is better than fish fed on commercial diet without application of biofluc ^33, 35, 36^. According to Azim ^34^ the nutritional quality of biofloc was appropriate at least for herbivorous and omnivorous fish species. In this case, the African catfish is categorized as omnivorous feeding habits ^35, 37^. 

It is clear that biofloc contributed to the growth and production of cultured organism as shown in this study. The basic principle of this technology is using the heterotrophic bacteria to manage the C: N ratio in the water media ^33, 38, 39^. However, biofloc not only contains the bacteria, but also is composed of other microorganisms including microalgae and zooplanktons as food for farmed fish or shrimps ^33^. According to Crab *et al*. ^40^ biofloc can be consumed and digested well by the shrimp and therefore possibly substitute for artificial commercial feed. Hence, application of biofloc into cultured system can increase feed efficiency up to 13% ^39^. For example, for feed efficiency of African catfish fed a commercial diet without biofloc was 89.83 % ^33^; it was increased up to 110.86% when the biofloc was applied as shown in this study.

In addition, according to Avnimelech ^38^, the addition of molasses as a source of carbon in aquaculture system can improve the C/N ratio waters and will further reduce inorganic nitrogen in the waters through increased growth of heterotrophic bacteria, where the heterotrophic bacteria will form a floc which can be fed by fish as feed source. Furthermore the C: N ratio of >10: 1 in the fish farming system is the optimum ratio to enhance the biofloc production and minimize the ammonia regeneration ^39^.

## Conclusion

The application of probiotic bacteria with different frequencies in the biofloc system had the significant effect on the growth performance, survival rate, and feed utilization of African catfish ( *Clarias gariepinus*). The higher growth performance and best feed utilization were recorded in the application of probiotic into biofloc system at 5-day intervals.

## Data availability

The data referenced by this article are under copyright with the following copyright statement: Copyright: © 2017 Putra I et al.

Data associated with the article are available under the terms of the Creative Commons Zero "No rights reserved" data waiver (CC0 1.0 Public domain dedication). 

Dataset 1: The total length, body weight and total feed consumed by fish at every experiment. 10.5256/f1000research.12438.d174980 ^41^
