# Supplementary material for: Growth performance and feed utilization of African catfish Clarias gariepinus fed a commercial diet and reared in the biofloc system enhanced with probiotic
Source: F1000Res. 2017 Aug 22;6:1545. [Version 1] doi: 10.12688/f1000research.12438.1 (PMC5585874; doi:10.12688/f1000research.12438.1)
Supplement: Peer review history

# Peer review: Referee response for version 1

**DOI:** 10.5256/f1000research.13468.r25289

**Published:** 2017-09-05

**Authors:** Rudy Agung Nugroho

**Affiliations:** 1 Animal Physiology, Development and Molecular Laboratory, Department of Biology, Faculty of Mathematic and Natural Science, Mulawarman University, Samarinda, Indonesia

1. Title is suitable and clearly defined the research that has been done.

 2. Abstract is well written and attract the reader. Please add information regarding the CFU of the bacillus (ex: 10 mL inoculants bacteria with density of *Bacillus* sp. about 5×10 ^10^ colony forming units (CFU) ....).

 3. Introduction: the introduction is well constructed and supported with current references.

 4. Methods: a) please be specific on the ethical guidelines that author's performed in this research. Which international/national ethical guidelines. b) Please explain why the author use 5 days interval in this research. Is there any previous/preliminary research?

 5. Results: Survival rate of B2 groups was only 65% (Raw data), any explanation?

 6. Discussion: Good Discussion.

 7. Conclusion: Well constructed summary.

 8. Reference: please revise reference #1 :  (Sahwan MF: The feed fish and shrimp. *Penebar Swadaya.* Jakarta. 1999. ), with original title and give translation. otherwise it cannot be traced.

I have read this submission. I believe that I have an appropriate level of expertise to confirm that it is of an acceptable scientific standard.

# Peer review: Referee response for version 1

**DOI:** 10.5256/f1000research.13468.r25287

**Published:** 2017-08-25

**Authors:** Hafrijal Syandri

**Affiliations:** 1 Department of Aquaculture, Faculty of Fisheries and Marine Science, Bung Hatta University , Padang, Indonesia

A comment for abstract: the fish was fed a commercial diet that contain 38% crude protein, twice a day at satiation, could you please show the time?

 A comment for the methodology:

 1. Please state the type of commercial feed used? floating or drowned feed?

 2. The weight gain of fish measured every 12 day for 60 days, please show the data weight gain every 12-days in bar diagram or line graph?

 3. Please explain the version (including city) of software used in statistical analysis?

I have read this submission. I believe that I have an appropriate level of expertise to confirm that it is of an acceptable scientific standard.
